# Desarrollo infantil en zonas pobres de Perú

**DOI:** 10.26633/RPSP.2017.71

**Published:** 2017-04-14

**Authors:** Adrián Alberto Díaz, Jorge Bacallao Gallestey, Rocío Vargas-Machuca, Roxana Aguilar Velarde

**Affiliations:** 1 Organización Panamericana de la Salud Organización Panamericana de la Salud Quito Ecuador Organización Panamericana de la Salud, Quito, Ecuador.; 2 Centro para la Investigación y Rehabilitación de las Ataxias Hereditarias Centro para la Investigación y Rehabilitación de las Ataxias Hereditarias La Habana Cuba Centro para la Investigación y Rehabilitación de las Ataxias Hereditarias, La Habana, Cuba.; 3 Equipo de Analistas royectistas y Consultores en Salud Lima Perú Equipo de Analistas, Proyectistas y Consultores en Salud, Lima, Perú.; 4 Academia Internacional de Cibernética Social Proporcionalista Academia Internacional de Cibernética Social Proporcionalista Bogotá Colombia Academia Internacional de Cibernética Social Proporcionalista, Bogotá, Colombia.

**Keywords:** Desarrollo infantil, pobreza, América Latina, Perú, Child development, poverty, Latin America, Peru, Desenvolvimento infantil, pobreza, América Latina, Peru

## Abstract

**Objetivos.:**

El objetivo del estudio fue mostrar la influencia de varios factores socioeconómicos en el desarrollo motor y del lenguaje de los niños menores de 5 años a partir del estudio de línea de base realizado en el marco del Programa Conjunto de Infancia, Seguridad Alimentaria y Nutrición, implementado por cinco agencias de Naciones Unidas en 65 distritos de los departamentos de Loreto, Ayacucho, Huancavelica y Apurímac de Perú.

**Métodos.:**

Se aplicaron modelos de regresión logística dicotómica para estimar la probabilidad de adquisición de los hitos motores y del lenguaje, y modelos de regresión polinomial para estimar el último hito y el número de hitos alcanzados. Se analizó la influencia de la educación de la madre, la ubicación de la vivienda (urbana o rural) y las necesidades básicas insatisfechas, sobre la diferencia entre el resultado alcanzado y el esperado para la edad.

**Resultados.:**

Los niños de áreas rurales, hijos de madres con baja escolaridad y pertenecientes a hogares con necesidades básicas insatisfechas exhiben valores más bajos en las dos áreas del desarrollo. El retraso se incrementa al aumentar el número de condiciones de riesgo.

**Conclusiones.:**

La evaluación del desarrollo y el acompañamiento a las familias en el proceso de crianza de los niños debe ser priorizado por los sistemas de salud y los programas sociales. Los instrumentos utilizados han sido sensibles a tres criterios de validación.

## Abstract (en)

**Objectives.:**

The objective of the study was to demonstrate the influence of several socioeconomic factors on the motor and language development of children under 5 from the baseline study conducted within the framework of the Joint Program for Children, Food Security, and Nutrition, implemented by five United Nations agencies across 65 districts in the departments of Loreto, Ayacucho, Huancavelica, and Apurímac, Peru.

**Methods.:**

Dichotomous logistic regression models were used to estimate the likelihood of achievement of motor and language milestones, while polynomial regression models were used to estimate the last milestone achieved and the number of milestones achieved. The study analyzes the influence that maternal education, urban vs. rural housing, and unmet basic needs have on the difference between actual results and expected results for age was analyzed.

**Results.:**

Children living in rural areas, those whose mothers had low educational attainment, and those from households with unmet basic needs exhibited poorer outcomes in the two areas of development assessed. As the number of risk factors increased, so did the developmental delay.

**Conclusions.:**

Evaluation of child development and follow-up of families during the child-rearing process should be prioritized by health systems and social programs. The instruments used were sensitive to three criteria for validation.

## RESUMO

**Objetivos.:**

Examinar a influência de vários fatores socioeconômicos no desenvolvimento motor e da linguagem de crianças menores de cinco anos com base no estudo de referência realizado como parte do Programa Conjunto de Segurança Alimentar e Nutricional de Crianças, implementado por cinco agências das Nações Unidas em 65 distritos das províncias de Loreto, Ayacucho, Huancavelica e Apurímac, no Peru.

**Métodos.:**

Foram empregados modelos de regressão logística dicotômica para estimar a probabilidade de aquisição dos marcos de desenvolvimento motor e de linguagem, e modelos de regressão polinomial para estimar o último marco e o número de marcos alcançados. Foi analisada a influência do nível educacional materno, zona de moradia (urbana ou rural) e necessidades básicas não satisfeitas na diferença entre o resultado alcançado e o esperado para a idade.

**Resultados.:**

As crianças de zonas rurais, filhos de mães com baixa escolaridade e pertencentes a domicílios com necessidades básicas não satisfeitas apresentam valores menores nas duas áreas do desenvolvimento estudadas. O atraso é maior à medida que aumenta o número de condições de risco.

**Conclusões.:**

A avaliação do desenvolvimento e o acompanhamento das famílias no processo de criação infantil devem ser priorizados pelos sistemas de saúde e programas sociais. Os instrumentos usados mostraram ser sensíveis aos três critérios de validação.

En diversos documentos y compromisos como la Cumbre Mundial en favor de la Infancia de 1990 (1) y la Declaración del Milenio de 2000 (2), los países han expresado la necesidad de situar a la niñez en el centro del proceso de desarrollo, han reconocido su derecho a participar en la construcción de una sociedad justa, pacífica y solidaria, y se han comprometido a alcanzar metas concretas. La mayoría de los países de la Región de las Américas lograron reducciones significativas de la mortalidad infantil y en menores de 5 años, lo que les permitió alcanzar el objetivo de desarrollo del milenio 4 previsto para 2015 (3). Paralelamente, desde principios de los noventa, se mantiene el debate sobre la necesidad de no limitar los programas y las políticas a la supervivencia, sino orientarlos también a pro-mover una adecuada calidad de vida de los que sobreviven. Esta polémica ha dado lugar a que, en años recientes, varios países de la Región, como Chile, Colombia y Perú, implementaran programas y proyectos dirigidos a reducir la mortalidad infantil y la desnutrición crónica y a promover el desarrollo infantil temprano (4–6).

Todo ello se sustenta en que las condiciones existentes durante la etapa prenatal y los primeros años de la vida explican gran parte de las variaciones en la salud del adulto y en el capital humano (7). El desarrollo infantil, caracterizado por la progresiva adquisición de estructuras y funciones durante las etapas tempranas de la vida, está influido por las condiciones socioeconómicas de los niños y sus familias (8–10).

Las intervenciones orientadas a promover el desarrollo infantil temprano afrontan la dificultad de establecer una línea basal y de evaluar sus impactos, pues las escalas disponibles para la evaluación del desarrollo infantil no son válidas en cualquier contexto social y cultural ni pueden aplicarse a nivel poblacional (11).

Distintos autores indican que el ordenamiento en la adquisición de capacidades motrices y del lenguaje responde a un patrón universal y que cada hito adquirido habilita o prepara la adquisición de nuevas habilidades y competencias como parte de un proceso continuo de desarrollo (12, 14). Por ejemplo, las habilidades motrices exhiben una sucesión predecible en los principales hitos en los primeros 24 meses de vida como, por ejemplo, sentarse sin apoyo, gatear, caminar con ayuda, pararse con apoyo, pararse solo o caminar solo. Idéntica situación se observa en el lenguaje expresivo, que también muestra una progresión lógica desde el balbuceo y la expresión de palabras hasta la construcción de frases (15). Sin embargo, esta sucesión no es un referente normativo, sino una guía para la evaluación del desarrollo, pues las condiciones socioeconómicas y socioculturales pueden provocar permutaciones en esas secuencias (14, 16, 17).

Desde 2007, en Perú se ha desarrollado un proceso de diseño y validación de un instrumento de evaluación del desarrollo de menores de 5 años aplicable en en-cuestas poblacionales, que fue aplicado en la línea de base del Programa Conjunto de Infancia, Seguridad Alimentaria y Nutrición, implementado por cinco agencias de Naciones Unidas en 65 distritos de los departamentos de Loreto, Ayacucho, Huancavelica y Apurímac. La población de dichos ámbitos, caracterizados por su elevada ruralidad, ascendía a unos 710 000 habitantes, mayoritariamente quechua-hablantes, y estaba expuesta a altas tasas de pobreza (que oscilaban entre 54,6 y 85,7% según las regiones) y a una inadecuada calidad de los servicios de educación, salud y extensión agropecuaria (18).

El objetivo de este estudio es conocer la influencia de diversos factores socioeconómicos en el desarrollo motor y el lenguaje de los niños menores de 5 años a partir del estudio de línea de base realizado en el marco del Programa Conjunto de Infancia, Seguridad Alimentaria y Nutrición.

## MATERIALES Y MÉTODOS

Durante los meses de enero y febrero de 2011, se realizó un estudio descriptivo transversal de las familias con niños menores de 5 años que residen en siete provincias de las regiones de Apurímac, Ayacucho, Huancavelica y Loreto. La muestra se diseñó en tres etapas con una selección aleatoria de conglomerados proporcionada por el Instituto Nacional de Estadística e Informática (INEI) (19). El tamaño de la muestra se calculó a partir de la prevalencia provincial de la desnutrición crónica infantil de 2007 (20), admitiendo un error de muestreo de 8%, con un nivel de confianza estadística de 95%, y una expectativa de no respuesta de 10%.

La información sobre el estado socioeconómico, la educación de la madre, la atención de salud, las inmunizaciones, el estado nutricional, la presencia de episodios de infecciones respiratorias agudas, y las enfermedades diarreicas agudas en niños menores de 5 años durante los 15 días anteriores al estudio, se obtuvo realizando entrevistas a las madres o cuidadores con el mismo método que emplea el INEI en sus encuestas nacionales (21).

Todos los niños se midieron y pesaron con equipos calibrados y a los niños entre 6 y 59 meses se midió la concentración de hemoglobina en una muestra de sangre capilar procesada con un hemoglobinómetro portátil (*HemoCue*®). Se evaluó el desarrollo motor de los menores de 24 meses y el desarrollo del lenguaje, en el grupo de 15 a 59 meses. No obstante la inclusión de variables nutricionales, el interés de este estudio se centra en los indicadores del desarrollo y en su dependencia con respecto a la condición socioeconómica representada por varios de sus marcadores.

Para evaluar el desarrollo motor, se utilizó la escala abreviada de desarrollo motor (EADM), compuesta por dibujos representativos de 6 hitos motores gruesos: sentarse sin apoyo, pararse con apoyo, gatear, caminar con apoyo, pararse sin apoyo y caminar sin apoyo, que se ha validado y utilizado en varios países (12, 22). La información generada con la EADM se obtuvo durante una entrevista realizada a la cuidadora principal del infante en el hogar, en la cual se le expusieron dibujos correspondientes a cada hito y se le solicitó que identificase aquel o aquellos que el niño ya hubiese alcanzado. Finalmente, se le pidió que identificara el dibujo correspondiente al último hito alcanzado.

De la entrevista se recabó información de las siguientes variables: a) edad en meses completos, b) un indicador binario para cada hito, que adquiere el valor 1 si la madre o cuidador asegura que el niño ya ejecuta la acción que representa la figura correspondiente al hito en cuestión, o si se constata que el niño ya es capaz de realizar dicha acción, y 0, en el caso contrario, y c) el último hito alcanzado.

Para evaluar el desarrollo del lenguaje se utilizó una prueba validada en el país (23), que se denominó Escala de Indicadores del Lenguaje del Niño Peruano (EIL) y que consta de los siguientes hitos enunciados según su orden temporal más probable: a) dice tres palabras o más independientemente de la calidad de su pronunciación; b) expresa verbalmente la acción que realiza el perro con una o dos palabras; c) responde a su nombre y apellido; d) reconoce las 5 figuras de una lámina; e) describe correctamente el uso de al menos cuatro de los objetos; f) repite correctamente al menos 6 de las palabras escuchadas; g) cuenta correctamente los 10 dedos, y h) reproduce las oraciones con un máximo de 2 errores en cada una. Esta información se obtuvo por evaluación directa del niño. Aunque este instrumento ha tenido un grado razonable de validación, la sucesión y el ordenamiento de los hitos es mucho más variable, tal como cabría esperar de habilidades mucho menos pautadas que las motrices.

A los efectos del análisis de datos, estas dos escalas (la de hitos motores y la de hitos del lenguaje) fueron la base de las variables dependientes o de respuesta. El cumplimiento de cada hito y la edad en meses del niño se utilizaron para estimar, mediante modelos de regresión logística dicotómica, los tiempos en que se alcanzan los hitos y el tiempo de transición de un hito a otro. Estas estimaciones se realizaron sólo para los hitos motores, cuya frecuencia de inversiones es razonablemente baja. Las dos variables de respuesta fundamentales se modelaron como función de la edad mediante un procedimiento de ajuste de curvas basado en el valor del coeficiente de determinación (R^2^).

La métrica del desarrollo consistió en calcular la diferencia D entre los valores observado y esperado de la edad para las dos variables de respuesta. Puesto que estas variables de respuesta se miden en meses decimales, la diferencia también se expresa en esa escala. Esta métrica se basa en el cálculo de desviaciones respecto a valores estimados (mediante modelos de regresión polinomial) del desarrollo de los niños de la población peruana estudiada, que se consideran referentes. Este enfoque es preferible al uso de referencias internacionales (que también se utilizaron para una descripción global), porque permite evaluar la influencia de los factores de estratificación elegidos sobre el desarrollo, libres del efecto de otros factores comunes a todos los niños peruanos, que pueden diferir de los que se utilizaron para definir esas normas internacionales, y porque tampoco depende del supuesto restrictivo de que la influencia de dichos factores es homogénea.

El segundo paso consistió en el cálculo de estadísticos descriptivos de la métrica según factores de estratificación de interés y en la construcción de indicadores binarios: la escolaridad de la madre (1, 6 o menos años de educación, 0, 7 años o más), las necesidades básicas insatisfechas (1, al menos una NBI, 0, ninguna NBI), y la condición urbano-rural (1, rural, 0, urbana). A partir de estos tres indicadores binarios se construyó el indicador sintético “número de condiciones de riesgo” (NCR), definido como la suma de los tres anteriores, que, a su vez, se codificó como 0, ninguna condición de riesgo, 1, una condición de riesgo, y 2, 2 ó más condiciones.

A fin de estimar los tiempos de adquisición de los hitos motores y de garantizar que los resultados son comparables con las normas internacionales, primero se identificaron los niños sin inversiones con respecto a la sucesión esperada de los hitos. Luego, se estratificaron en 5 grupos (P_1_, P_2_, …, P_6_) definidos del siguiente modo: P_i_: la población de los niños que han alcanzado el hito i y el i−1, pero no el i+1 ni ninguno de los hitos posteriores. Las medias de las edades en los grupos P_i_ (i = 1, 2, …, 6) permitieron estimar la edad en la cual se adquiere el hito i. Finalmente, se sustrajo la media de la edad en P_i−1_ de la media de la edad en P_i_ y se obtuvo una estimación del tiempo medio en transitar entre hitos sucesivos. Para el resto de los análisis ya descritos se trabajó con todos los niños, tuvieran o no inversiones en la secuencia temporal de los hitos. Los análisis se realizaron con la versión 18 del programa estadístico SPSS y el Anthro 3.2.2. El protocolo del estudio fue aprobado por el Comité de Ética de la Asociación Benéfica PRISMA de Perú.

## RESULTADOS

En total, se evaluaron 1 176 niños menores de 5 años (589 niños y 587 niñas) (cuadro 1). En relación con el desarrollo motor, se evaluaron 313 niños menores de dos años de los cuales dos se excluyeron del análisis porque no habían cumplido el hito 1. La media de la edad de los niños fue 12,9 meses, con un mínimo de 0,13 meses y un máximo de 24,0 meses. El cuadro 2 muestra el número y el porcentaje de casos con inversiones de los hitos (por encima de la diagonal principal) y de los que se ajustan al orden esperado (por debajo de la diagonal). Las inversiones más frecuentes fueron: la del hito 3 con respecto al hito 4, que se presentó en 13% de los niños, y la del hito 2 con respecto al hito 3, que se observó en 11,1% de los casos. El 47,1% de los niños mostró alguna inversión en la sucesión temporal de los seis hitos ya descrita.

**CUADRO 1.** Distribución de los niños evalaudos según sus características y las pruebas de desarrollo, Lima, 2011

Características		Con EADM (niños de 0–23 meses)	Con EIL (niños de 15–59 meses)
Grupos de edad (meses)	0–11	102	–
	12–23	211	172
	24–35	0	229
	36–47	0	249
	48–59	0	213
	Total	313	863
Sexo	Hombre	166	423
	Mujer	147	440
	Total	313	863
Área geográfica	Urbano	129	343
	Rural	184	520
	Total	313	863
Pobreza por necesidades básicas insatisfechas	Sí	168	431
	No	145	432
	Total	313	863
Educación de la madre (años)	0–6	153	460
	7 o más	160	403
	Total	313	863

EADM: escala abreviada de desarrollo motor.

EIL: escala de indicadores de lenguaje.

Debido a la heterogeneidad en la adquisición de los hitos, se eligió como variables de respuesta al último hito y al número de hitos alcanzados, que coindicen en ausencia de inversiones. Considerando R^2^ como criterio de elección, en ambos casos el modelo cúbico arrojó el mejor ajuste. Los modelos fueron los siguientes:

UHM = 3,063 – 0,475* edad + 0,061*      edad^2^ – 0,002* edad^3^NHM = 0,123 + 0,109* edad + 0,027*      edad^2^ – 0,001* edad^3^

donde UHM es el último hito motor alcanzado y NHM, el número de hitos motores alcanzados.

Los residuos de estos modelos representan el retraso (o adelanto) de un niño con respecto a un valor de referencia estimado a partir de poblaciones de niños peruanos con sus mismas características. Si estos residuos se estratificaran por variables como la escolaridad de la madre, las necesidades básicas insatisfechas en el hogar y el área urbana o rural, mostrarían la influencia de dichas variables sobre el desarrollo motor.

El cuadro 3 contiene las medias y las desviaciones estándar de los residuos del último hito motor y del número de hitos motores alcanzados según los factores de estratificación corregidas para puntajes z de talla para la edad y hemoglobina ajustada para la altura. Hay diferencias (pequeñas, pero sistemáticamente en la misma dirección) en el desarrollo motor para ambos indicadores respecto a la educación de la madre y al área geográfica, y más discretas respecto a las NBI. Los hijos de madres con 7 o más años de educación, los que vivían en hogares sin NBI y los de áreas urbanas tenían un desarrollo motor mayor de lo esperado para la edad. Lo contrario ocurre con los hijos de madres con educación primaria o menos, con los que viven en hogares que tienen NBI y con los del área rural. Cuando los tres indicadores se integran en un recuento de condiciones de riesgo, las diferencias son más ostensibles: los niños sin condiciones de riesgo tenían un desarrollo mucho mayor que el de los que tenían 2 o más condiciones de riesgo.

**CUADRO 2.** Número (porcentaje) de casos con inversiones (por encima de la diagonal principal) y no inversiones (por debajo de la diagonal principal) en los hitos motores de los niños estudiados, Lima, 2011

Hitos^a^	2	3	4	5	6
2		35 (11,1)	28 (8,9)	18 (5,7)	17 (5,4)
3	280 (88,9)		41 (13,0)	5 (1,69	6 (1,9)
4	287 (91,1)	274 (87,0)		2 (0,6)	3 (1,0)
5	297 (94,3)	310 (98,4)	313 (99,4)		14 (4,4)
6	298 (95,6)	309 (98,1)	312 (99,0)	301 (95,6)	

a Se excluye el hito 1 del análisis porque sólo se detectaron dos niños que no lo habían vencido.

**CUADRO 3.** Medias y desviaciones estándar (en meses decimales) de los residuos ajustados para la edad del último hito motor y del de hitos alcanzados según las variables de estratificación corregidas por puntajes z de talla/edad y hemoglobina corregida por altura,^a^ Lima, 2011

Variables de estratificación	Estratos (n)	Último hito motor		Número de hitos motores	
		Media	Desviación estándar	Media	Desviación estándar
Educación de la madre (años)	0 a 6 (151)	-0,22	1,31	-0,12	0,94
	7 o más (161)	0,20	1,08	0,11	0,92
Necesidades básicas insatisfechas	Una o más (167)	-0,08	1,29	-0,02	0,92
	Sin (145)	0,09	1,11	0,02	0,95
Área geográfica	Rural (184)	-0,23	1,33	-0,14	0,96
	Urbana (128)	0,33	0,98	0,20	0,86
Número de condiciones de riesgo	0 (64)	0,35	0,91	0,23	0,88
	1 (78)	0,21	1,11	0,14	0,94
	2 o más (170)	-0,21	1,30	-0,05	0,93

a El número de condiciones de riesgo se obtiene como el conteo de factores desfavorables entre los que contiene la tabla: educación de la madre de 6 años o menos, existencia de alguna NBI y vivienda en el ámbito rural.

**CUADRO 4.** Edad en meses decimales para los percentiles 25, 50 y 75 de las referencias de la Organización Mundial de la Salud (OMS) y de los resultados de este estudio, Lima, 2011

Hitos	Percentiles					
	25		50		75	
	OMS	Perú	OMS	Perú	OMS	Perú
2	6,6	6,0	7.4	7,5	8,4	8,0
3	7,4	8,0	8,3	10,0	9,3	13,0
4	9,0	10,0	10,0	13,0	11,0	15,0
5	9,7	12,0	10,8	15,5	12,0	18,0
6	12,0	16,0	13,1	18,0	14,4	23,0

En el cuadro 4 se presentan los cuartiles de la distribución de la edad en meses para el logro del hito de referencias de la OMS y la población de estudio. Destaca que, mientras que en el segundo hito los niños peruanos se comportaban de forma ligeramente diferente de los de la referencia internacional, esta brecha aumentaba con la progresión de hitos. Por ejemplo, para el hito 5, las diferencias se encontraban entre los 3 y 6 meses para los percentiles 25 y 75, respectivamente. Esto indica un efecto acumulativo en la edad de presentación del hito.

Para el estudio de los hitos del lenguaje se dispuso de una muestra de 863 niños con edad promedio de 34,2 meses, un mínimo de 15 y un máximo de 60 meses. El número de casos sin inversiones fue de 401 (46,5%).

El ajuste de los modelos para estimar el último hito y el número de hitos alcanzados arrojó resultados similares a los hitos motores y condujeron igualmente a la elección del modelo cúbico:

UHL = 0,157 – 0,072* edad + 0,008*      edad^2^ – 0,00009* edad^3^NHL = 2,392 – 0,218* edad + 0,009*      edad^2^ – 0,00008* edad^3^

donde UHL es el último hito del lenguaje alcanzado y NHL, el número de hitos del lenguaje alcanzados.

Los resultados del cuadro 5 muestran la influencia de los factores vinculados con la pobreza sobre el desarrollo del lenguaje. Para ambos indicadores, los niños de áreas rurales, cuyas madres tenían 6 o menos años de educación y vivían en hogares con alguna NBI, presentaban el mayor retraso en la adquisición de los hitos de lenguaje. Las brechas eran aún más marcadas que en el desarrollo motor y se mantenían después de corregir para el puntaje Z de talla/edad y la hemoglobina ajustada por altura.

**CUADRO 5.** Medias y desviaciones estándar (en meses decimales) de los residuos ajustados para la edad del último hito del lenguaje y el de hitos alcanzados según variables de estatificación, corregidas por puntajes z de talla/edad y hemoglobina corregida por altura,^a^ Lima, 2011

Variables de estratificación	Estratos (n)	Último hito de lenguaje		Número de hitos de lenguaje	
		Media	Desviación estándar	Media	Desviación estándar
Educación de la madre (años)	0 a 6 años (462)	-0,28	1,90	-0,28	1,57
	7 o más (400)	0,33	1,89	0,32	1,59
Necesidades básicas insatisfechas	Con una o más (167)	-0,06	1,93	-0,11	1,61
	Sin (145)	0,06	1,90	0,11	1,62
Área geográfica	Rural (523)	-0,23	1,92	-0,24	1,59
	Urbana (339)	0,34	1,86	0,36	1,59
Número de condiciones de riesgo	0 (226)	0,30	1,90	0,56	0,88
	1 (290)	-0,04	1,92	0,21	0,94
	2 o más (246)	-0,14	1,88	-0,07	0,93

a El número de condiciones de riesgo se obtiene como el recuento de factores desfavorables entre los que contiene la tabla: educación de la madre de 6 años o menos, existencia de alguna NBI y vivienda en el ámbito rural.

## DISCUSIÓN

Pocos temas tienen tanta presencia en la bibliografía contemporánea como el crecimiento lineal de los niños, sus determinantes y consecuencias, lo cual está siendo priorizado por las políticas sociales de la mayoría de los países de la Región (24).

Menor atención se ha prestado al desarrollo infantil y, peor aún, se han trasladado conceptos del crecimiento lineal al neurodesarrollo, como traduce el uso de la desafortunada expresión metafórica “ventana de oportunidades” para referirse al período que transcurre durante toda la etapa prenatal y los primeros 24 meses de vida extrauterina. Si bien es cierto que durante estos primeros 1 000 días se acumula casi todo el retraso en la talla, no puede afirmarse lo mismo del neurodesarrollo. Pese a la abrumadora evidencia de que éste es, en efecto, el momento más oportuno para intervenir, el error por exceso consiste en pasar por alto que, después de ese período, cualquier desatención implica afectaciones serias del desarrollo y que hay un espectro bien amplio de acciones no limitadas a los dos primeros años de vida para favorecerlo y estimularlo (25). El término “ventana” trans-mite la idea errónea de que toda acción posible o efectiva queda restringida a ese período, lo cual está siendo cuestionado por diversos autores (26, 27). El origen del error es considerar que el crecimiento lineal es *per se* el objeto de las acciones, cuando en realidad es sólo un marcador de otros componentes de la salud, la nutrición y el desarrollo, que sí definen metas que se deben alcanzar (24).

Los efectos de la pobreza sobre la salud de los niños han generado grandes polémicas (28). Las interpretaciones de sus mecanismos se orientan en dos sentidos: uno netamente biologicista, centrado en los modos y estilos de vida de los pobres, y otro que pone más acento en los procesos sociales que los generan. Por otra parte, todos los factores que tienen un impacto sobre la salud de los niños lo ejercen también sobre su desarrollo.

Hay una fuerte asociación entre la situación socioeconómica y la salud. Los países con mayores brechas socioeconómicas tienen también peores indicadores de salud. Este “mapeo” de las desigualdades sociales sobre las sanitarias es prácticamente universal e incluye a los países con mayor desarrollo económico (29, 30).

Las privaciones en el orden socioeconómico se acompañan de un amplio ran-go de incompetencias en las esferas física y mental del desarrollo. Recíprocamente, la imposibilidad de los niños para alcanzar su potencial biológico de desarrollo, junto con las causas que la generan, desempeñan un papel importante en la transmisión intergeneracional de la pobreza. El incremento de la educación materna y la mejora en las condiciones socioeconómicas generan un amplio espectro de consecuencias positivas, que se hacen sentir en la salud, la nutrición y el desarrollo físico y mental (31).

Varios estudios, transversales y longitudinales, documentan la relación de la pobreza y de sus factores asociados, tanto con el crecimiento, como con el desarrollo de los niños en las esferas cognitiva y motriz (32, 33).

Aunque no están del todo claros los mecanismos que vinculan la pobreza, sus manifestaciones y secuelas con el retraso en el desarrollo cognitivo de los niños, se considera que las repercusiones de las privaciones socioeconómicas y de los bajos niveles de educación de las familias que las padecen tienen un impacto negativo en sus facultades expresivas, que a su vez favorecen el retraso en el desarrollo del vocabulario debido al efecto acumulativo de la exposición a ambientes carentes de estímulos para el aprendizaje, como muestran estudios realizados en diferentes países, incluido Perú (34–36).

El presente estudio hace hincapié en la importancia de medir el desarrollo infantil a nivel poblacional y aporta dos instrumentos para ese fin, uno aplicable a la medición del desarrollo motor en niños de 6 a 24 meses y otro, a la medición del desarrollo del lenguaje para niños entre 12 y 59 meses. Además, incluye varias instancias de validación por criterios de dichos instrumentos, que consisten en mostrar que ambos son sensibles a los efectos de variables que usualmente se asocian con la pobreza, como la baja escolaridad de la madre, la existencia de NBI y el ámbito rural. En él se propone utilizar, por añadidura, un instrumento simplificado que intenta capturar peso de las competencias familiares para el cuidado y la estimulación respecto de su desarrollo.

Una limitación metodológica consiste en que se ha realizado a partir de un diseño transversal, lo cual obliga a utilizar un recurso de interpolación, que permite obtener estimaciones de los indicadores de desarrollo como función de la edad y evaluar el efecto de los factores de estratificación sobre las desviaciones relativas a los valores esperados para la edad.

Se han puesto de manifiesto notables diferencias vinculadas con los tres factores asociados con la pobreza, tanto en el desarrollo motor como en el desarrollo cognitivo. Respecto al desarrollo motor, si bien se observó alguna inversión en la sucesión temporal de los hitos, ello ya ha sido notificado por otros autores y no invalidaría la utilización de la escala (17). En este sentido, aunque los instrumentos deben validarse en otras poblaciones, se ha mostrado que son sensibles a tres criterios de validación.

La principal novedad metodológica de este estudio es la utilización de un procedimiento de interpolación basado en la aplicación de modelos polinomiales para estimar los hitos motores alcanzados como función de la edad. El hecho de haber demostrado que el retraso en el desarrollo se incrementa con el número de factores de predisposición proporciona un criterio de focalización para las intervenciones dirigidas a mejorar el desarrollo motor y cognitivo de los niños.

### Agradecimiento.

Los autores reconocen y agradecen las valiosas contribuciones del Dr. Ernesto Pollitt† en el proceso de definición de los instrumentos simplificados para la valoración del desarrollo infantil a nivel poblacional.

#### Financiación.

El presente estudio se realizó en el marco del Programa Conjunto de Infancia, Seguridad Alimentaria y Nutrición, financiado por el Fondo para el Desarrollo de los Objetivos del Milenio (F-ODM).

#### Declaración.

Las opiniones expresadas en este manuscrito son responsabilidad del autor y no reflejan necesariamente los criterios ni la política de la *RPSP/PAJPH* y/o de la OPS.
